# One-Year Incidences of Venous Thromboembolism, Bleeding, and Death in Patients With Lung Cancer (Cancer-VTE Subanalysis)

**DOI:** 10.1016/j.jtocrr.2022.100392

**Published:** 2022-08-08

**Authors:** Nobuyasu Awano, Tetsuya Okano, Riken Kawachi, Masaru Matsumoto, Tetsuya Kimura, Atsushi Takita, Mari S. Oba, Hideo Kunitoh

**Affiliations:** a Department of Respiratory Medicine, Japanese Red Cross Medical Center, Tokyo, Japan; b Department of Respiratory Medicine, Nippon Medical School Chiba Hokusoh Hospital, Inzai, Japan; c Department of Respiratory Surgery, Nihon University School of Medicine, Tokyo, Japan; d Department of Pulmonary Medicine and Oncology, Graduate School of Medicine, Nippon Medical School, Tokyo, Japan; e Primary Medical Science Department, Daiichi Sankyo Co., Ltd., Tokyo, Japan; f Data Intelligence Department, Daiichi Sankyo Co., Ltd., Tokyo, Japan; g Department of Medical Statistics, Toho University, Tokyo, Japan; h Department of Clinical Data Science, Clinical Research & Education Promotion Division, National Center of Neurology and Psychiatry, Tokyo, Japan; i Department of Medical Oncology, Japanese Red Cross Medical Center, Tokyo, Japan

**Keywords:** Hemorrhage, Lung neoplasms, Mortality, Risk, Thromboembolism

## Abstract

**Introduction:**

This subanalysis aimed to provide real-world data on venous thromboembolism (VTE) from patients with lung cancer in the Cancer-VTE Registry.

**Methods:**

The primary outcome was the number of baseline VTE events in patients with lung cancer. The 1-year cumulative incidences of symptomatic VTE; composite VTE (symptomatic and incidental VTE requiring treatment); bleeding; cerebral infarction, transient ischemic attack, and systemic embolic events; and all-cause death were calculated. Clinical trial registration: UMIN000024942.

**Results:**

The study enrolled a total of 2377 patients with lung cancer; of these, 119 (5.0%) had VTE (six [0.3%], symptomatic, and 113 [4.8%], asymptomatic) and 14 (0.6%) had pulmonary embolism at baseline. During the follow-up period (mean, 337.7 d), the incidence was 0.6% for symptomatic VTE, 1.8% for composite VTE, 1.5% for bleeding events, 1.3% for cerebral infarction, transient ischemic attack, and systemic embolism, and 19.1% for all-cause death. Composite VTE frequency did not vary by anticancer drug type. Patients with (versus without) VTE at baseline had higher hazard ratios (HRs) for composite VTE (unadjusted HR: 5.29; Gray test *p* < 0.001) and symptomatic VTE (unadjusted HR: 4.89; Gray test *p* = 0.007). Patients with VTE at baseline had higher HRs for bleeding events (unadjusted HR: 3.27; Gray test *p* = 0.010) and all-cause death (unadjusted HR: 2.73; log-rank test *p* < 0.001) than patients without. In multivariable analysis, patients with baseline VTE prevalence and Eastern Cooperative Oncology Group Performance Status of 2 had increased composite VTE risk during cancer therapy. There were no other risk factors for composite VTE.

**Conclusions:**

Our findings emphasize the importance of VTE screening at cancer diagnosis.

## Introduction

Venous thromboembolism (VTE) is a hypercoagulable state that can include both deep vein thrombosis and pulmonary embolism (PE).^1^ Stagnation of blood flow, vascular endothelial damage, and hypercoagulability owing to surgery, trauma, hospitalization, and the use of central venous catheters are risk factors for VTE.^1^^,^^2^ Recently, it has been widely recognized that patients with cancer are particularly susceptible to VTE.^3^^,^^4^ Across all cancers, the risk of VTE is elevated approximately sevenfold compared with patients without cancer.^5^ The risk of VTE is particularly high within the first few months of cancer diagnosis and in patients with distant metastases.^5^

Lung cancer is the most common type of malignancy in Japan and is the leading cause of death among patients with cancer.^6^ This suggests that VTE may be a fundamental health care problem in this patient cohort. The bidirectional association between cancer and VTE and the underlying pathophysiology linking the two conditions are well established.^7^ Several risk factors for VTE among patients with cancer have been described and can be broadly categorized as tumor-related, treatment-related, and patient-related factors.^8^^,^^9^ Tumor-related risk factors for VTE include the type and location of the primary lesion.^3^

Among patients with lung cancer, VTE is more common in patients with adenocarcinoma. In addition, the VTE risk is particularly high in *ALK*-positive lung cancer.^10^ As for treatment-related factors, surgery, catheterization, and prolonged bed rest have been associated with VTE risk.^11^ Furthermore, there have been reports that therapeutic agents such as bevacizumab, platinum, and immune checkpoint inhibitors (ICIs) may increase the risk of thrombosis.^11^, ^12^, ^13^, ^14^, ^15^, ^16^, ^17^ In terms of patient-related factors, racial characteristics seem to be important. Epidemiologic studies have suggested that Asian patients with cancer have a threefold to fivefold lower incidence of symptomatic VTE compared with Caucasian patients^18^^,^^19^; however, there are few large prospective studies in Japanese patients with cancer.

The current lack of VTE incidence rates in Japanese patients with lung cancer, particularly from extensive cohort studies, is just one of many major evidential knowledge gaps worldwide relating to VTE in cancer,^2^ and additional data are urgently needed to inform diagnostic and management decisions. Thus, the large-scale (including almost 10,000 patients), prospective Japanese Cancer-VTE Registry was initiated among patients with solid tumors before starting treatment. In addition, the Registry explored the incidence and risk factors of clinical events during 1 year of follow-up.^20^, ^21^, ^22^ The objective of the present subanalysis of data from the Japanese Cancer-VTE Registry was to investigate outcomes relating to VTE, bleeding, embolic events, and all-cause death after 1 year of follow-up in the cohort of patients with lung cancer. In addition, this subanalysis aimed to investigate the potential risk factors associated with these events.

## Materials and Methods

### Design of the Cancer-VTE Registry

This was a nationwide, multicenter clinical registry conducted in accordance with the Declaration of Helsinki and the Ethical Guidelines for Medical Science Studies on Human Subjects by the Japanese Ministry of Education, Culture, Sports, Science, and Technology and the Ministry of Health, Labour and Welfare. All patient data were anonymized.

The study included a prespecified, observational prospective cohort analysis over 1 year of follow-up, with all management decisions made at the treating physician’s discretion. The ethics committees of each participating institution approved the study protocol and all related documentation. Overall, patients with colorectal, lung, stomach, pancreatic, breast, or gynecologic cancer from 170 Japanese medical institutions were followed up from March 2017 to February 2020. The study design has been fully described in previous publications.^21^^,^^22^ The focus of this analysis was the cohort of patients with lung cancer.

### Patients

In brief, the inclusion criteria relating to patients with lung cancer were as follows: age of 20 years or older; lung tumor stages IB to IV; life expectancy more than or equal to 6 months; Eastern Cooperative Oncology Group (ECOG) performance status (PS) of 0 to 2; and provision of written informed consent for participation. Outpatients and hospitalized patients were eligible for study inclusion, with enrollment occurring before any planned cancer therapy initiation. Previous cancer therapy for a primary tumor followed by stable disease for 6 months or more before disease progression or recurrence was allowed.

Baseline VTE screening requirements were such that patients with a D-dimer concentration after cancer diagnosis of less than or equal to 1.2 μg/mL were regarded as non-VTE^23^ and were not required to undergo VTE screening. VTE screening 2 months before enrollment was an eligibility criterion for all other patients. The processes conformed to current Japanese guidelines,^24^ and the preferred screening modality was venous ultrasonography of the lower extremity; this could be substituted by computed tomography (CT) angiography of the lower extremity. At the physician’s discretion, confirmation of PE was by means of contrast CT or other diagnostic imaging tests.

### Study Outcomes

The primary outcome for this analysis was the number of baseline VTE events in patients with lung cancer, and the 1-year cumulative incidences of symptomatic VTE; composite VTE (symptomatic VTE events and incidental [asymptomatic] VTE events requiring treatment); bleeding (major or clinically relevant nonmajor bleeding); cerebral infarction, transient ischemic attack (TIA), and systemic embolic event (SEE); and all-cause death were calculated. Incidental VTE requiring treatment was defined as one of the following: (1) incidentally discovered asymptomatic VTE for which treatment was clinically indicated and initiated during the follow-up period; or (2) asymptomatic VTE detected at baseline screening that was left untreated and for which therapy was initiated during the follow-up period because clinically so indicated (e.g., owing to exacerbation). The incidence of each event was evaluated according to the presence of potential risk factors, including cancer subtype, recurrence, metastasis, stage, PS, actual treatment, monotherapy or combination therapy, and a class of systemic therapy. All events were adjudicated by independent committees, including neurologists and cardiovascular and VTE specialists.

### Statistical Methods

Categorical variables were tabulated using n (%) and continuous variables using mean, SD, and median. Time-to-event rates were calculated using the cumulative incidence function for each event of interest. Between-group differences according to baseline VTE status were explored using the Gray test (for VTE, bleeding, and cerebral infarction, TIA, and SEE) or the log-rank test (for all-cause death). Incidences were also calculated as population proportions at 1 year of follow-up.

Univariable analyses were conducted to investigate factors correlated with the presence or absence of VTE at baseline using logistic regression models. Univariable and multivariable analyses were conducted to investigate factors associated with the occurrence of composite VTE during the follow-up period using the Fine and Gray models, with all-cause death as a competing event. In multivariable analysis, the following explanatory variables (adjustment factors) were used: presence or absence of VTE at the time of registration, cancer stage (IB–II and III–IV), ECOG PS (0–1 and 2), cancer therapy method (surgery, chemotherapy, and radiotherapy), cancer subtypes (SCLC, adenocarcinoma, squamous cell carcinoma, others), and anticoagulation therapy. “Chemotherapy” in this study includes treatments with target-based drugs, angiogenesis inhibitors, and ICIs, including cytotoxic chemotherapy. All statistical analyses were conducted using SAS software version 9.4 (SAS Institute Inc., Cary, NC).

## Results

### Baseline Characteristics

Of the patients enrolled in the Cancer-VTE Registry, 2377 had lung cancer. Of the patients with lung cancer, 119 (5.0%) had VTE at baseline (Table 1). The mean age of patients with lung cancer was 69.4 years, and most patients (1690 of 2377 [71.1%]) were of male sex. No tests were conducted for the significance level of differences between patient characteristics according to VTE at baseline. Descriptive comparisons revealed that higher proportions of patients with lung cancer and VTE at baseline were older and more likely to be aged 65 years or older. These patients were also more frequently of female sex and had a lower body mass index. Regarding comorbidities, a greater proportion of patients with lung cancer and VTE at baseline had a history of VTE, gastrointestinal bleeding, decreased renal function (creatinine clearance ≤ 50 mL/min), anemia (hemoglobin [Hb] < 10 g/dL), and white blood cell (WBC) count greater than 11 × 10^9^/L, compared with patients with lung cancer without VTE. Of the 119 patients with VTE at baseline, 42 (35.3%) were receiving an anticoagulant (warfarin or direct oral anticoagulant) at the time of enrollment. D-dimer exceeding the published cutoff value of 1.2 μg/mL was more common among patients with lung cancer with VTE than those without VTE.^23^

**Table 1:** Baseline Demographics, Medical and Laboratory Characteristics, and Tumor-Associated Characteristics of Patients With Lung Cancer in the Cancer-VTE Registry

Characteristic	Patients With Lung Cancer (n = 2377 [100%])	With VTE at Baseline (n = 119 [5.0%])	Without VTE at Baseline (n = 2258 [95.0%])
Male sex, n (%)	1690 (71.1)	65 (54.6)	1625 (72.0)
Age, y			
Mean (SD)	69.4 (9.6)	73.4 (7.3)	69.1 (9.7)
≥65, n (%)	1773 (74.6)	111 (93.3)	1662 (73.6)
BMI, kg/m^2^			
Mean (SD)	22.5 (3.5)	22.0 (3.6)	22.5 (3.5)
≥25, n (%)	506 (21.3)	17 (14.3)	489 (21.7)
Smoking history, n (%)			
Yes	319 (13.4)	13 (10.9)	306 (13.6)
No (previous smoking)	1575 (66.3)	66 (55.5)	1509 (66.8)
Never	483 (20.3)	40 (33.6)	443 (19.6)
Presence of complication, n (%)			
Hypertension	1020 (42.9)	58 (48.7)	962 (42.6)
Atrial fibrillation	99 (4.2)	1 (0.8)	98 (4.3)
Liver dysfunction	54 (2.3)	2 (1.7)	52 (2.3)
Peptic ulcer	66 (2.8)	6 (5.0)	60 (2.7)
Medical history, n (%)			
VTE	19 (0.8)	13 (10.9)	6 (0.3)
Cerebral infarction	107 (4.5)	4 (3.4)	103 (4.6)
Intracranial hemorrhage	39 (1.6)	0 (0.0)	39 (1.7)
Gastrointestinal bleeding	28 (1.2)	4 (3.4)	24 (1.1)
Bed rest for 4 d or more	35 (1.5)	7 (5.9)	28 (1.2)
DOAC or warfarin use,^a^ n (%)	139 (5.8)	42 (35.3)	97 (4.3)
Laboratory test values D-dimer, μg/mL			
Mean (SD)	1.9 (5.0)	9.0 (10.5)	1.5 (4.2)
Median	0.7	5.1	0.7
>1.2, n (%)	573 (24.1)	109 (91.6)	464 (20.5)
CrCL, mL/min			
Mean (SD)	75 (26)	67 (26)	76 (26)
≤50, n (%)	323 (13.6)	25 (21.0)	298 (13.2)
Platelet count, ×10^9^/L			
Mean (SD)	262 (88)	259 (93)	262 (87)
≥350, n (%)	316 (13.3)	15 (12.6)	301 (13.3)
Hb, g/dL			
Mean (SD)	13.4 (1.6)	12.4 (1.8)	13.5 (1.6)
<10, n (%)	68 (2.9)	9 (7.6)	59 (2.6)
WBC count, ×10^9^/L			
Mean (SD)	7.2 (2.6)	8.3 (4.4)	7.2 (2.5)
>11, n (%)	162 (6.8)	19 (16.0)	143 (6.3)
Cancer subtype, n (%)			
SCLC	279 (11.7)	14 (11.8)	265 (11.7)
Non-SCLC	2004 (84.3)	102 (85.7)	1902 (84.2)
Adenocarcinoma	1251 (52.6)	76 (63.9)	1175 (52.0)
Squamous cell carcinoma	558 (23.5)	19 (16.0)	539 (23.9)
NOS	195 (8.2)	7 (5.9)	188 (8.3)
Other	94 (4.0)	3 (2.5)	91 (4.0)
Primary cancer, n (%)	2221 (93.4)	110 (92.4)	2111 (93.5)
With lymph node metastasis, n (%)	1387 (58.4)	85 (71.4)	1302 (57.7)
With distant metastasis, n (%)	778 (32.7)	83 (69.7)	695 (30.8)
Cancer stage, n (%)			
IB	459 (19.3)	8 (6.7)	451 (20.0)
II	518 (21.8)	11 (9.2)	507 (22.5)
III	599 (25.2)	15 (12.6)	584 (25.9)
IV	801 (33.7)	85 (71.4)	716 (31.7)
ECOG PS, n (%)			
0	1404 (59.1)	23 (19.3)	1381 (61.2)
1	849 (35.7)	72 (60.5)	777 (34.4)
2	124 (5.2)	24 (20.2)	100 (4.4)

*Note:* Percentages illustrated in the table were calculated on the basis of the total in each column unless otherwise specified.

BMI, body mass index; CrCL, creatinine clearance; DOAC, direct oral anticoagulant; ECOG, Eastern Cooperative Oncology Group; Hb, hemoglobin; NOS, not otherwise specified; PS, performance status; VTE, venous thromboembolism; WBC, white blood cell count.

a Oral anticoagulant treatment that started before enrollment.

The tumor-associated baseline variables are summarized in Table 1. Most patients with lung cancer (2004 of 2377 [84.3%]) had NSCLC (primarily adenocarcinoma). In addition, advanced disease (metastases to the lymph nodes or distant sites, stage IV cancer, and ECOG PS of 2) and adenocarcinoma were more frequent in the subgroup with VTE than in the subgroup without VTE.

### VTE at Baseline

A summary of the types of VTE at baseline in patients with lung cancer is found in Table 2. Of 119 of 2377patients (5.0%) with lung cancer diagnosed with VTE at baseline, six (0.3%) had symptomatic VTE, 113 (4.8%) had asymptomatic VTE, and 14 (0.6%) had PE. Asymptomatic distal deep vein thrombosis accounted for 100 of 119 patients (84.0%) with VTE.

**Table 2:** Summary of VTE Prevalence at Baseline in Patients With Lung Cancer

n (%)	All	Symptomatic	Asymptomatic
All VTE	119 (5.0)	6 (0.3)	113 (4.8)
PE (with or without DVT)	14 (0.6)	3 (0.1)	11 (0.5)
DVT (with or without PE)	114 (4.8)	4 (0.2)	110 (4.6)
Proximal DVT	12 (0.5)	2 (0.1)	10 (0.4)
Distal DVT	102 (4.3)	2 (0.1)	100 (4.2)

*Note:* Data in the table were calculated on the basis of N = 2377.

DVT, deep vein thrombosis; PE, pulmonary embolism; VTE, venous thromboembolism.

Univariable analysis of factors correlated with VTE prevalence at baseline is found in Supplementary Table 1. The risk factors associated with VTE incidence at baseline were female sex, older age (≥65 y), cancer stage IV, lymph node metastasis, distant metastasis, ECOG PS of 1 and 2, history of VTE, bed rest for 4 days or more, D-dimer greater than 1.2 μg/mL, Hb less than 10 g/dL, WBC count greater than 11 × 10^9^/L, and creatinine clearance less than or equal to 50 mL/min.

### Incidence of Events During Follow-Up

During a mean follow-up period of 337.7 days, among the patients with lung cancer, the incidence of VTE and other events was 0.6% for symptomatic VTE, 1.8% for composite VTE, 1.5% for bleeding events, 1.3% for cerebral infarction, TIA, and SEE, and 19.1% for all-cause death (Table 3). Approximately 90% of all-cause deaths were primary cancer-related deaths. The incidence of all events was higher in patients with VTE at baseline than in those without VTE.

**Table 3:** Incidence of Events During the Follow-Up Period

Event	Patients With Lung Cancer (n = 2377)		With VTE at Baseline (n = 119)		Without VTE at Baseline (n = 2258)	
	Patients With Events, n	Incidence (95% CI)	Patients With Events, n	Incidence (95% CI)	Patients With Events, n	Incidence (95% CI)
Symptomatic VTE	15	0.6 (0.4–1.0)	3	2.5 (0.5–7.2)	12	0.5 (0.3–0.9)
Incidental VTE requiring treatment	31	1.3 (0.9–1.8)	7	5.9 (2.4–11.7)	24	1.1 (0.7–1.6)
Composite VTE^a^	43	1.8 (1.3–2.4)	9	7.6 (3.5–13.9)	34	1.5 (1.0–2.1)
Bleeding^b^	35	1.5 (1.0–2.0)	5	4.2 (1.4–9.5)	30	1.3 (0.9–1.9)
Cerebral infarction, TIA, and SEE	32	1.3 (0.9–1.9)	3	2.5 (0.5–7.2)	29	1.3 (0.9–1.8)
All-cause death	455	19.1 (17.6–20.8)	47	39.5 (30.7–48.9)	408	18.1 (16.5–19.7)

CI, confidence interval; SEE, systemic embolic event; TIA, transient ischemic attack; VTE, venous thromboembolism.

a A composite of symptomatic VTE events and incidental VTE events requiring treatment.

b Included major bleeding and clinically relevant nonmajor bleeding events.

**Figure 1:**
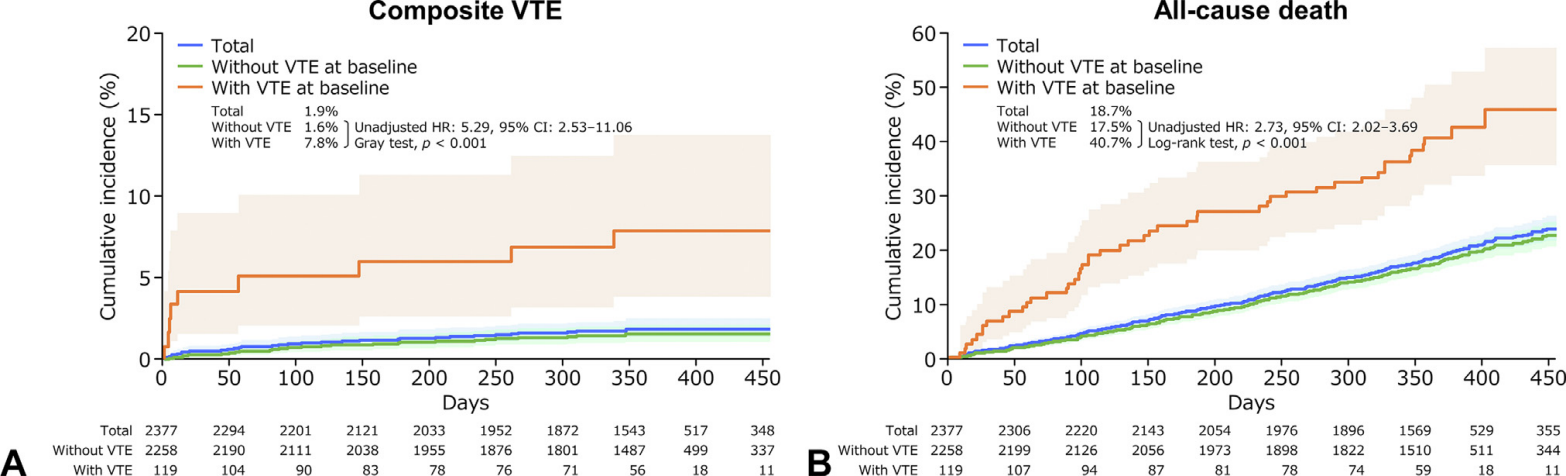
Cumulative incidence of events (time-to-event analysis). (*A*) Composite VTE and (*B*) all-cause death. *p* values were calculated using (*A*) the Gray test or (*B*) the log-rank test. Lightly shaded areas represent 95% CIs. CI, confidence interval; HR, hazard ratio; VTE, venous thromboembolism.

The cumulative incidences of composite VTE and all-cause death according to VTE at baseline are found in Figures 1*A* and *B*, respectively. Patients with VTE at baseline had a higher hazard ratio (HR) for composite VTE (unadjusted HR = 5.29, 95% confidence interval [CI]: 2.53–11.06, Gray test *p <* 0.001) and for all-cause death (unadjusted HR = 2.73, 95% CI: 2.02–3.69, log-rank test *p <* 0.001) than patients without VTE. Supplementary Figure 1*A* to *C* reveals the cumulative incidence of symptomatic VTE, bleeding events, and cerebral infarction, TIA, and SEE in patients with lung cancer with and without VTE at baseline. Patients with VTE at baseline had higher HRs for symptomatic VTE (unadjusted HR = 4.89, 95% CI: 1.38–17.33, Gray test *p =* 0.007) and bleeding events (unadjusted HR = 3.27, 95% CI: 1.26–8.46, Gray test *p =* 0.010) compared with patients without VTE. There were no differences in cerebral infarction, TIA, and SEE incidences between patients with and without VTE.

### Event Occurrence According to Cancer Subtype, Tumor-Related Variables, and Cancer Therapy

The incidence of composite VTE during the follow-up period according to cancer subtype, tumor-related variables, and cancer therapy is found in Supplementary Table 2. Of the patients with NSCLC, those with the adenocarcinoma subtype had a numerically higher composite VTE incidence than squamous cell carcinoma. The incidence of VTE events tended to be higher in patients with more advanced cancer stages and higher ECOG PS. Regarding cancer therapies, the incidences of composite VTE were 1.5% for patients receiving surgery, 2.6% for those undergoing chemotherapy, and 3.1% for radiotherapy, respectively.

### Event Occurrence According to Systemic Chemotherapy

Event occurrence according to systemic chemotherapy (anticancer drug therapy) is found in Table 4. The incidence of VTE events (symptomatic and composite VTE) was numerically similar among patients receiving each systemic chemotherapy, except that those receiving angiogenesis inhibitors had numerically lower incidences of symptomatic VTE, incidental VTE requiring treatment, composite VTE, and bleeding. Concerning tyrosine kinase inhibitors (TKIs), *EGFR* TKIs were used by 257 patients, and 10 of these patients (3.9%) developed composite VTE. *ALK* TKIs were used by 36 patients, of which none presented with composite VTE.

**Table 4:** Incidence of Events Occurring During the Follow-Up Period According to Systemic Therapy

Event		Taxanes (n = 361)	TKI (n = 292)	*EGFR* TKI (n = 257)	*ALK* TKI (n = 36)	ICIs (n = 378)	Platinum (n = 1047)	Pyrimidine Fluoride (n = 256)	Other Cytotoxic Anticancer Agents (n = 780)	Angiogenesis Inhibitors (n = 139)
Symptomatic VTE	n (%)	3 (0.8)	2 (0.7)	2 (0.8)	0 (0.0)	6 (1.6)	11 (1.1)	4 (1.6)	8 (1.0)	0 (0.0)
	95% CI	0.2–2.4	0.1–2.5	0.1–2.8	0.0–9.7	0.6–3.4	0.5–1.9	0.4–4.0	0.4–2.0	0.0–2.6
Incidental VTE requiring treatment	n (%)	6 (1.7)	8 (2.7)	8 (3.1)	0 (0.0)	10 (2.6)	20 (1.9)	8 (3.1)	15 (1.9)	0 (0.0)
	95% CI	0.6–3.6	1.2–5.3	1.4–6.0	0.0–9.7	1.3–4.8	1.2–2.9	1.4–6.1	1.1–3.2	0.0–2.6
Composite VTE^a^	n (%)	8 (2.2)	10 (3.4)	10 (3.9)	0 (0.0)	15 (4.0)	29 (2.8)	11 (4.3)	21 (2.7)	0 (0.0)
	95% CI	1.0–4.3	1.7–6.2	1.9–7.0	0.0–9.7	2.2–6.5	1.9–4.0	2.2–7.6	1.7–4.1	0.0–2.6
Bleeding^b^	n (%)	9 (2.5)	3 (1.0)	3 (1.2)	0 (0.0)	10 (2.6)	20 (1.9)	4 (1.6)	12 (1.5)	1 (0.7)
	95% CI	1.1–4.7	0.2–3.0	0.2–3.4	0.0–9.7	1.3–4.8	1.2–2.9	0.4–4.0	0.8–2.7	0.0–3.9
Cerebral infarction, TIA, and SEE	n (%)	7 (1.9)	3 (1.0)	3 (1.2)	0 (0.0)	5 (1.3)	18 (1.7)	2 (0.8)	14 (1.8)	3 (2.2)
	95% CI	0.8–4.0	0.2–3.0	0.2–3.4	0.0–9.7	0.4–3.1	1.0–2.7	0.1–2.8	1.0–3.0	0.4–6.2
All-cause death	n (%)	117 (32.4)	43 (14.7)	37 (14.4)	6 (16.7)	116 (30.7)	269 (25.7)	31 (12.1)	208 (26.7)	37 (26.6)
	95% CI	27.6–37.5	10.9–19.3	10.3–19.3	6.4–32.8	26.1–35.6	23.1–28.5	8.4–16.7	23.6–29.9	19.5–34.8

CI, confidence interval; ICI, immune checkpoint inhibitor; SEE, systemic embolic event; TIA, transient ischemic attack; TKI, tyrosine kinase inhibitor; VTE, venous thromboembolism.

a A composite of symptomatic VTE events and incidental VTE events requiring treatment.

b Included major bleeding and clinically relevant nonmajor bleeding events.

### Risk Factors for Composite VTE

The results of multivariable analysis of risk factors for composite VTE during the follow-up period are found in Table 5. Although the number of events was small, significant factors associated with composite VTE during the follow-up period were ECOG PS (HR = 3.94, 95% CI: 1.74–8.91, *p <* 0.001) and VTE prevalence at baseline (HR = 4.75, 95% CI: 1.99–11.38, *p =* 0.001). There were no significant differences across cancer types, but the HR of adenocarcinoma (1.39, 95% CI: 0.52–3.73, *p =* 0.516) was greater than the HR for squamous cell carcinoma (0.40, 95% CI: 0.10–1.60, *p =* 0.193). Surgery, chemotherapy, and radiotherapy were not risk factors for the development of composite VTE.

**Table 5:** Univariable and Multivariable Analyses of Risk Factors for Composite VTE During the Follow-Up Period

Items		n	Events, n (%)	Univariable			Multivariable		
				HR	95% CI	*p* Value	HR	95% CI	*p* Value
Cancer stage	IB/II	977	11 (1.1)	1.00	—	—	1.00	—	—
	III/IV	1400	32 (2.3)	2.07	1.04–4.10	0.037	1.80	0.77–4.19	0.173
ECOG PS	0 or 1	2253	33 (1.5)	1.00	—	—	1.00	—	—
	2	124	10 (8.1)	5.85	2.87–11.89	<0.001	3.94	1.74–8.91	<0.001
VTE at baseline	No	2258	34 (1.5)	1.00	—	—	1.00	—	—
	Yes	119	9 (7.6)	5.29	2.53–11.06	<0.001	4.75	1.99–11.38	0.001
Cancer subtype	SCLC	279	6 (2.2)	1.00	—	—	1.00	—	—
	Adenocarcinoma	1251	31 (2.5)	1.13	0.47–2.70	0.787	1.39	0.52–3.73	0.516
	Squamous cell carcinoma	558	4 (0.7)	0.33	0.09–1.15	0.082	0.40	0.10–1.60	0.193
	Other^a^	289	2 (0.7)	0.31	0.06–1.55	0.154	0.39	0.06–2.42	0.314
Oral anticoagulant treatment^b^	No	2238	42 (1.9)	1.00	—	—	1.00	—	—
	Yes	139	1 (0.7)	0.39	0.05–2.82	0.348	0.16	0.02–1.21	0.076
Surgery^c^	No	1321	28 (2.1)	1.00	—	—	1.00	—	—
	Yes	1056	15 (1.4)	0.66	0.35–1.23	0.190	1.52	0.66–3.47	0.324
Chemotherapy^c^	No	796	11 (1.4)	1.00	—	—	1.00	—	—
	Yes	1581	32 (2.0)	1.46	0.74–2.91	0.277	0.97	0.37–2.55	0.949
Radiotherapy^c^	No	1903	33 (1.7)	1.00	—	—	1.00	—	—
	Yes	474	10 (2.1)	1.20	0.60–2.44	0.607	1.22	0.60–2.51	0.581

CI, confidence interval; ECOG, Eastern Cooperative Oncology Group; HR, hazard ratio; PS, performance status; VTE, venous thromboembolism.

a Other than SCLC, adenocarcinoma, and squamous cell carcinoma.

b Oral anticoagulant treatment that started before enrollment.

c Cancer therapy before the occurrence of the composite VTE event.

## Discussion

This study provides real-world data on the clinical status and VTE incidence among Japanese patients with lung cancer during a 1-year follow-up period from before starting cancer therapy to after VTE screening. VTE screening at the time of cancer diagnosis revealed a VTE prevalence of 5.0%. The cumulative incidences of symptomatic and composite VTE during the follow-up period were 0.6% and 1.8%, respectively.

The previously reported overall study results of the Cancer-VTE Registry revealed that VTE prevalence at the time of cancer diagnosis was 5.9% and symptomatic VTE at the 1-year follow-up period was 0.5%.^20^^,^^21^ In our subanalysis of patients with lung cancer, the incidence of VTE at baseline and during follow-up was as low as in the main study. Differences in race, VTE screening, disease severity, and treatment characteristics may explain the low VTE incidence in the present study.

Regarding race, previous studies on non-Japanese patients with lung cancer reported incidences of VTE of 1.3% to 2.6% (within 2 y in a U.S. registry study), 39.2 per 1000-person years (in a U.K. database study), and 8.2% (within 1 y in a French database study), with an extensive range in frequencies between studies.^25^, ^26^, ^27^ As for VTE screening, the incidence of VTE during the follow-up period was lower in our study than that in the study by Kenmotsu et al.^28^ (patients with stage IV lung cancer in this subanalysis, 2.7%; previous study, 14.1%), although the VTE prevalence at baseline was similar in both studies (patients with stage IV lung cancer in this subanalysis, 10.6%; previous study, 11.3%). In the study by Kenmotsu et al.,^28^ VTE screening was performed at enrollment and follow-up. Conversely, VTE screening was not performed during the follow-up period in our study. We consider that these differences in study design affected the difference in VTE incidence rate during the follow-up period.

Concerning disease severity, in a previous study, patients with metastatic or locally advanced lung cancer who were not receiving anticoagulants had a cumulative VTE incidence of 24.1% (147 of 611) during 24 weeks after starting cancer therapy.^28^ Another retrospective study reported that VTE developed in 71 of 682 Japanese patients (10.4%) with newly diagnosed lung cancer who were tested for VTE at admission and included 97.1% of patients with stages III to IV disease, compared with 58.9% in our analysis.^29^ Compared with previous studies, our study included more patients with less severe cancer. Concerning treatment characteristics, a lower percentage of patients underwent surgery in previous studies than in our study (10.8%–19.6% versus 44.5%).^25^^,^^26^ The present study included more patients with good PS in the early stage who were eligible for surgery. Thus, the low incidence of VTE in the present study could be attributed to varying races, VTE screening methods, disease severity, and treatment characteristics.

The risk factors for VTE at baseline obtained from our univariable analysis were female sex, age, cancer stage, lymph node metastasis, distant metastasis, ECOG PS, history of VTE, bed rest for 4 days or more, increased D-dimer level, low Hb, and high WBC count. The results were broadly consistent with a previous retrospective study of Asian patients with lung cancer complicated with VTE at baseline, which identified similar risk factors (e.g., sex, age, tumor pathologic type, tumor stage, and serum levels of WBC, Hb, platelets, and D-dimer).^30^ In patients with these risk factors for VTE, it may be advisable to perform D-dimer measurement and VTE screening before starting cancer therapy. The Khorana score is well known to be a VTE predictor, and the score is calculated using the cancer site, WBC count, Hb level, platelet count, and body mass index.^31^

In the present study, low Hb and high WBC results are consistent with the Khorana score. Conversely, the platelet level did not seem to be associated with baseline VTE in this study. Our study also identified the risk factors for VTE during the follow-up period as follows: patients with baseline VTE prevalence and ECOG PS of 2. Although the patient background and design of this study were not in complete agreement with previous studies, the present results support several reports from Asia revealing ECOG PS is a risk factor for VTE.^32^^,^^33^ These results also suggest that VTE, even if asymptomatic before cancer therapy, was associated with a higher incidence of each event, including death, during multiple cancer therapies, which is also consistent with the main study results.^20^

Remarkable progress has been made in the development of therapeutic agents for lung cancer in recent years, and the range of systemic therapies available to physicians has expanded.^34^^,^^35^ Some anticancer drugs, platinum drugs,^11^, ^12^, ^13^, ^14^ ICIs,^16^ and anti–vascular endothelial growth factor agents^15^ reportedly increase thrombus formation and predispose the patient to develop VTE. The effect of each drug class on VTE expression has been studied and reported.^11^, ^12^, ^13^, ^14^, ^15^, ^16^, ^17^ Nevertheless, to our knowledge, no reports have evaluated the effect of a broad category of anticancer drugs on VTE expression in a single study, as we have accomplished in the present study. Unlike previous studies,^15^ in this study, there were no incidences of VTE with vascular endothelial growth factor inhibition. Although the reasons are unclear, we speculate that physicians in this study had identified whether their patients were at high risk for these events and prescribed the drugs appropriately. The incidence of VTE was high among patients treated with ICIs compared with other class therapeutic agents in this study, but it was lower than that of the previous studies.^16^ At the time of this study, almost no patients were receiving chemotherapy plus ICI, which is now the global standard of care, and most patients were receiving ICI after second-line treatment. It has been reported that patients with *ALK*-positive lung cancer are four times more likely to have VTE than patients with other types of lung cancer.^10^ In our study, we could not analyze how many *ALK*-positive patients with lung cancer at baseline developed VTE, but none of the 36 patients who used *ALK* inhibitors during the follow-up period developed VTE. The frequency of VTE did not vary by type of anticancer drug in our study. Nevertheless, this was a large-scale prospective study that included patients with various background factors, and some potentially relevant risk factors may not have been accounted for. Thus, on the basis of the present study results, we cannot conclude an association between use of particular anticancer drugs and VTE.

This study has several limitations. First, this study can have potential selection bias because the eligibility restriction of cancer type, stage, and anticancer drug selection depends on the attending physician's judgment. In addition, differences in whether the anticancer drug was used as first-line therapy or second-line therapy and whether it was used in combination with an ICI may have influenced VTE development. Second, this was an observational study and did not require routine VTE screening with contrast-enhanced CT or lower extremity venous echocardiography during the follow-up period, which could have resulted in missed VTE. Third, treatment of VTE at the time of enrollment was at the discretion of the attending physician. Anticoagulants (warfarin or direct oral anticoagulants) may have been used for unrelated comorbidities such as atrial fibrillation, but it may also have been used for the treatment of asymptomatic VTE, which could have led to fewer VTE events in the follow-up period. Finally, the follow-up period was short at 1 year; thus, it is possible that VTE could not be adequately identified as it can become a cancer complication at the later stages of the disease.

In conclusion, this is the first large-scale, long-term study investigating VTE in patients with lung cancer in the current clinical setting in Japan. Among patients with lung cancer enrolled in the Cancer-VTE Registry, those with VTE before cancer therapy were at higher risk of VTE during cancer therapy. This finding underlines the importance of VTE screening at the time of cancer diagnosis.

## CRediT Authorship Contribution Statement

Nobuyasu Awano: Conceptualization; Writing – original draft; Writing – review & editing. Tetsuya Okano: Conceptualization; Writing – review & editing. Riken Kawachi: Conceptualization; Writing – review & editing. Masaru Matsumoto: Conceptualization; Writing – review & editing. Tetsuya Kimura: Conceptualization; Writing – original draft; Writing – review & editing. Atsushi Takita: Conceptualization; Writing – original draft; Writing – review & editing. Mari S Oba: Conceptualization; Formal analysis; Writing – original draft; Writing – review & editing. Hideo Kunitoh: Supervision; Conceptualization; Writing – original draft; Writing – review & editing.

## Data Sharing Statement

The anonymized data underlying the results presented in this manuscript may be made available to researchers on submission of a reasonable request to the corresponding author. The decision to disclose the data will be made by the corresponding author and the funder, Daiichi Sankyo Co., Ltd. The data disclosure can be requested for 36 months from the article publication.
