# Supplementary material for: One-Year Incidences of Venous Thromboembolism, Bleeding, and Death in Patients With Lung Cancer (Cancer-VTE Subanalysis)
Source: JTO Clin Res Rep. 2022 Aug 8;3(9):100392. doi: 10.1016/j.jtocrr.2022.100392 (PMC9460508; doi:10.1016/j.jtocrr.2022.100392)
Supplement: Supplementary material [file mmc1.docx]

**Supplemental Table 1** Univariable analysis of factors correlated with VTE prevalence at baseline

| Factor |  | n | Events,  n (%) | OR | 95% CI | *p* value |
| --- | --- | --- | --- | --- | --- | --- |
| Sex | Male | 1690 | 65 (3.8) | 1.00 | - | - |
|  | Female | 687 | 54 (7.9) | 2.13 | 1.47–3.10 | <0.001 |
| Age, years | <65 | 604 | 8 (1.3) | 1.00 | - | - |
|  | ≥65 | 1773 | 111 (6.3) | 4.98 | 2.41–10.26 | <0.001 |
| Cancer stage | IB/II | 977 | 19 (1.9) | 1.00 | - | - |
|  | III | 599 | 15 (2.5) | 1.30 | 0.65–2.57 | 0.459 |
|  | IV | 801 | 85 (10.6) | 5.99 | 3.61–9.94 | <0.001 |
| Lymph node metastasis | No | 990 | 34 (3.4) | 1.00 | - | - |
|  | Yes | 1387 | 85 (6.1) | 1.84 | 1.22–2.76 | 0.003 |
| Distant metastasis | No | 1599 | 36 (2.3) | 1.00 | - | - |
|  | Yes | 778 | 83 (10.7) | 5.19 | 3.47–7.75 | <0.001 |
| Occurrence of cancer | Primary | 2221 | 110 (5.0) | 1.00 | - | - |
|  | Recurrence | 156 | 9 (5.8) | 1.18 | 0.58–2.37 | 0.652 |
| ECOG PS | 0 | 1404 | 23 (1.6) | 1.00 | - | - |
|  | 1 | 849 | 72 (8.5) | 5.56 | 3.45–8.97 | <0.001 |
|  | 2 | 124 | 24 (19.4) | 14.41 | 7.86–26.44 | <0.001 |
| BMI, Kg/m^2^ | <18.5 | 272 | 18 (6.6) | 1.27 | 0.75–2.16 | 0.367 |
|  | 18.5 to <25 | 1594 | 84 (5.3) | 1.00 | - | - |
|  | ≥25 | 506 | 17 (3.4) | 0.63 | 0.37–1.06 | 0.083 |
| Cancer subtype | SCLC | 279 | 14 (5.0) | 1.00 | - | - |
|  | Non-SCLC | 2004 | 102 (5.1) | 1.02 | 0.57–1.80 | 0.959 |
|  | Adenocarcinoma | 1251 | 76 (6.1) | 1.22 | 0.68–2.20 | 0.498 |
|  | Squamous cell carcinoma | 558 | 19 (3.4) | 0.67 | 0.33–1.35 | 0.261 |
|  | NOS | 195 | 7 (3.6) | 0.71 | 0.28–1.78 | 0.459 |
|  | Other | 94 | 3 (3.2) | 0.62 | 0.18–2.22 | 0.467 |
| History of VTE | No | 2358 | 106 (4.5) | 1.00 | - | - |
|  | Yes | 19 | 13 (68.4) | 46.03 | 17.16–123.48 | <0.001 |
| Bed rest for 4 days or more | No | 2342 | 112 (4.8) | 1.00 | - | - |
|  | Yes | 35 | 7 (20.0) | 4.98 | 2.13–11.64 | <0.001 |
| D-dimer, μg/mL | ≤1.2 | 1705 | 6 (0.4) | 1.00 | - | - |
|  | >1.2 | 573 | 109 (19.0) | 66.52 | 29.06–152.29 | <0.001 |
| Platelet count,  × 10^9^/L | <350 | 1961 | 94 (4.8) | 1.00 | - | - |
|  | ≥350 | 316 | 15 (4.7) | 0.99 | 0.57–1.73 | 0.971 |
| Hb, g/dL | ≥10 | 2209 | 100 (4.5) | 1.00 | - | - |
|  | <10 | 68 | 9 (13.2) | 3.22 | 1.55–6.67 | 0.002 |
| WBC count,  × 10^9^/L | ≤11 | 2115 | 90 (4.3) | 1.00 | - | - |
|  | >11 | 162 | 19 (11.7) | 2.99 | 1.77–5.04 | <0.001 |
| CrCL, mL/min | >50 | 1951 | 84 (4.3) | 1.00 | - | - |
|  | ≤50 | 323 | 25 (7.7) | 1.87 | 1.17–2.96 | 0.008 |

Abbreviations: BMI, body mass index; CI, confidence interval; CrCL, creatinine clearance; ECOG, Eastern Cooperative Oncology Group; Hb, hemoglobin; NOS, not otherwise specified; OR, odds ratio; PS, performance status; SCLC, small cell lung cancer; VTE, venous thromboembolism; WBC, white blood cell.

**Supplemental Table 2** Incidence of composite VTE during the follow-up period by cancer subtype, tumor-related variables, and cancer therapy

|  | All | Composite VTE | |
| --- | --- | --- | --- |
|  | n (%) | n (%) | 95% CI |
| Total | 2377 (100.0) | 43 (1.8) | 1.3–2.4 |
| Cancer subtype |  |  |  |
| SCLC | 279 (11.7) | 6 (2.2) | 0.8–4.6 |
| Non-SCLC | 2004 (84.3) | 36 (1.8) | 1.3–2.5 |
| Adenocarcinoma | 1251 (52.6) | 31 (2.5) | 1.7–3.5 |
| Squamous cell carcinoma | 558 (23.5) | 4 (0.7) | 0.2–1.8 |
| NOS | 195 (8.2) | 1 (0.5) | 0.0–2.8 |
| Other | 94 (4.0) | 1 (1.1) | 0.0–5.8 |
| Tumor-related variables |  |  |  |
| Cancer stage |  |  |  |
| IB | 459 (19.3) | 4 (0.9) | 0.2–2.2 |
| II | 518 (21.8) | 7 (1.4) | 0.5–2.8 |
| III | 599 (25.2) | 10 (1.7) | 0.8–3.0 |
| IV | 801 (33.7) | 22 (2.7) | 1.7–4.1 |
| ECOG PS |  |  |  |
| 0 | 1404 (59.1) | 20 (1.4) | 0.9–2.2 |
| 1 | 849 (35.7) | 13 (1.5) | 0.8–2.6 |
| 2 | 124 (5.2) | 10 (8.1) | 3.9–14.3 |
| Cancer therapy |  |  |  |
| Surgery | 1057 (44.5) | 16 (1.5) | 0.9–2.4 |
| Chemotherapy | 1590 (66.9) | 41 (2.6) | 1.9–3.5 |
| Radiotherapy | 479 (20.2) | 15 (3.1) | 1.8–5.1 |
| No cancer therapy | 153 (6.4) | 0 (0) | 0.0–2.4 |

Abbreviations: CI, confidence interval; ECOG, Eastern Cooperative Oncology Group; NOS, not otherwise specified; PS, performance status; SCLC, small cell lung cancer; VTE, venous thromboembolism.

**Supplemental Figure 1**

**A**

**
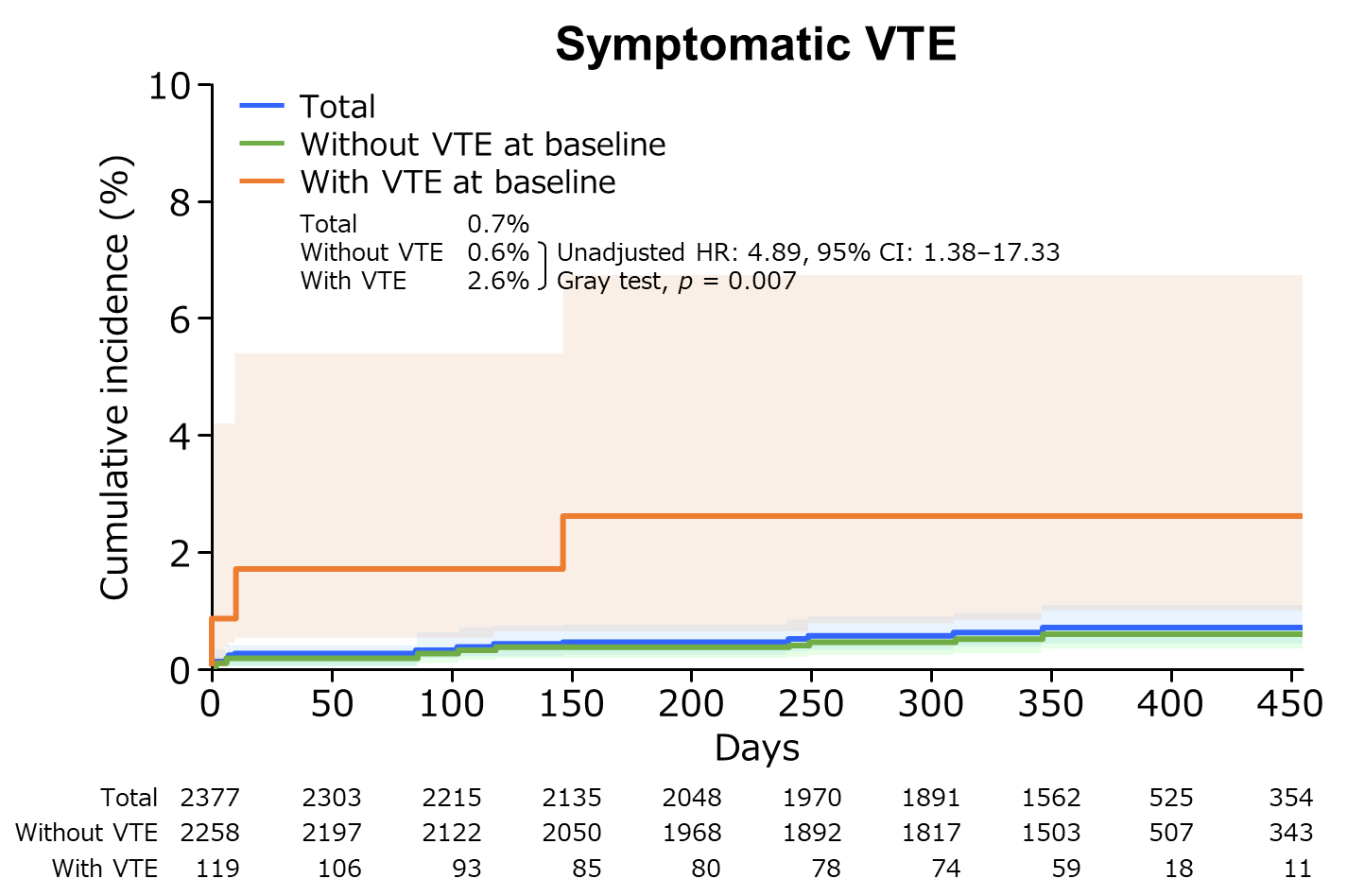
**

**B**


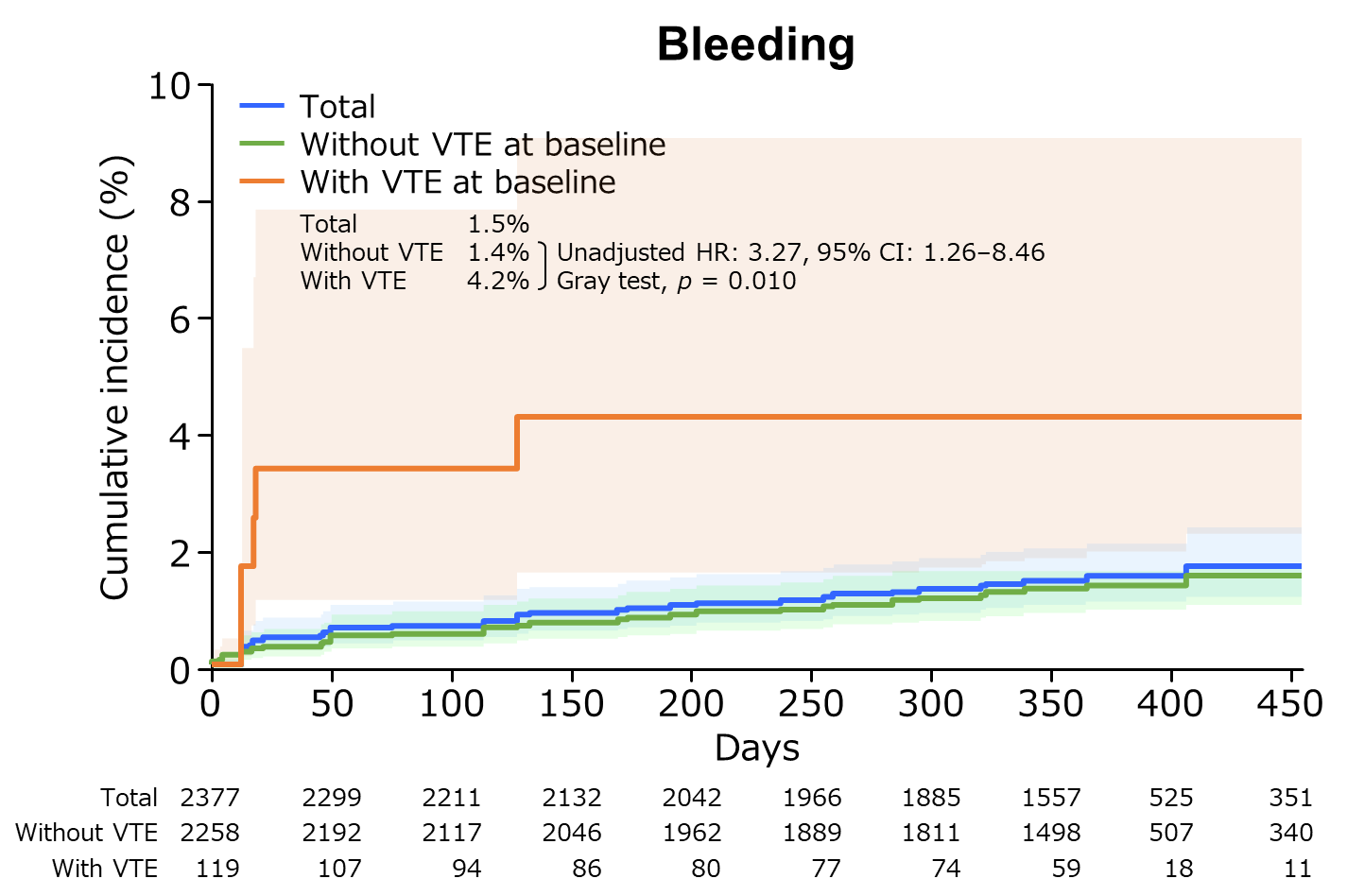


**C**
**
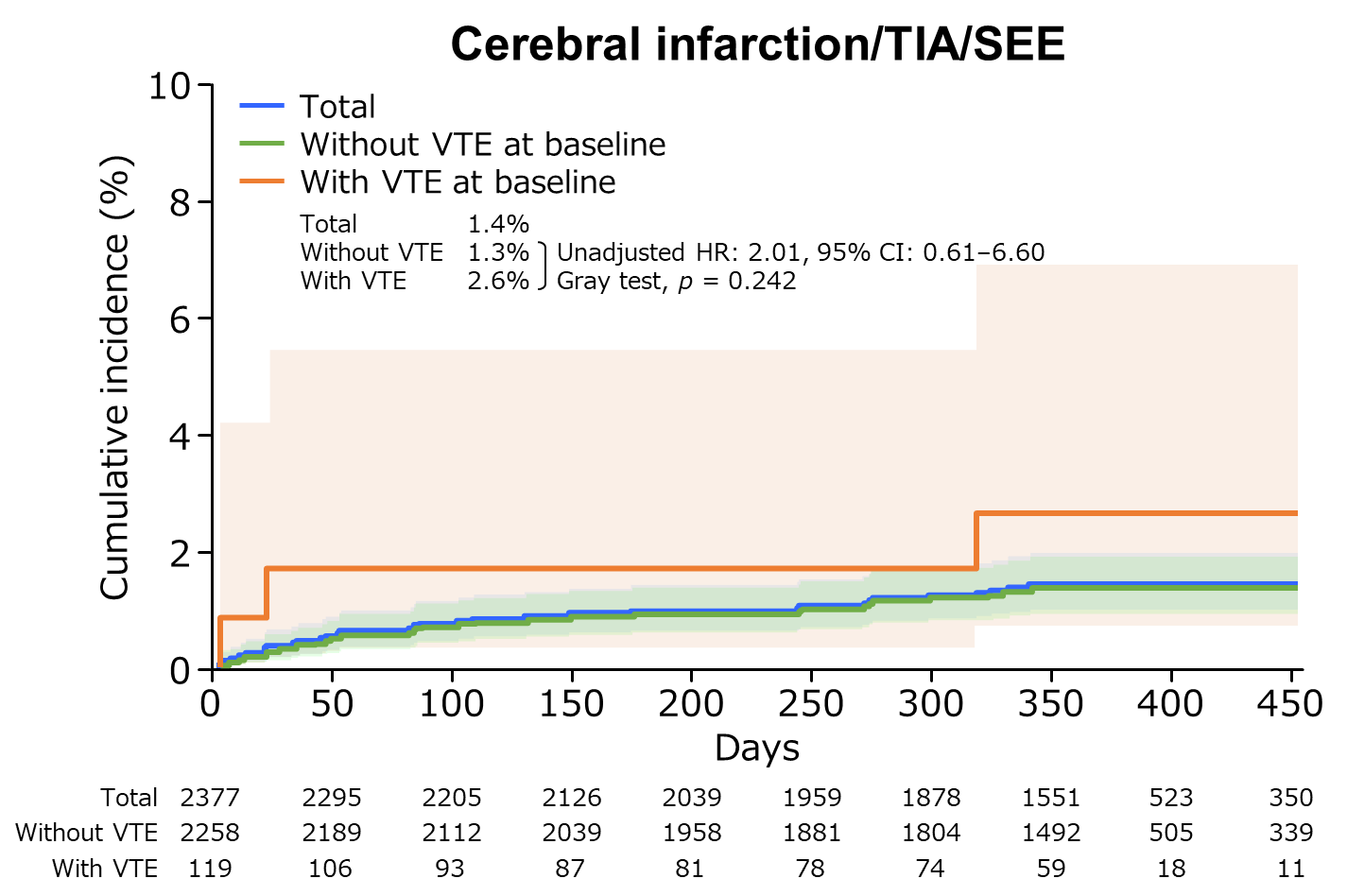
**

**Supplemental Figure 1** Cumulative incidence of events (time-to-event analysis). (A) Symptomatic VTE, (B) bleeding events, and (C) cerebral infarction, TIA, and SEE.
*P* values were calculated using the Gray test. Lightly shaded areas represent 95% CIs. Abbreviations: CI, confidence interval; HR, hazard ratio; SEE, systemic embolic event; TIA, transient ischemic attack; VTE, venous thromboembolism.
